# Investigating best practices of district-wide physical activity programmatic efforts in US schools– a mixed-methods approach

**DOI:** 10.1186/s12889-018-5889-4

**Published:** 2018-08-30

**Authors:** Christina D. Economos, Megan P. Mueller, Nicole Schultz, Julie Gervis, Gabrielle F. Miller, Russell R. Pate

**Affiliations:** 1 ChildObesity180, Gerald J. and Dorothy R. Friedman School of Nutrition Science and Policy, Tufts University, Boston, MA USA; 2 Friedman School of Nutrition Science and Policy, Tufts University, Boston, MA USA; 3 Centers for Disease Control and Prevention, Atlanta, GA USA; 4 Arnold School of Public Health, University of South Carolina, Columbia, SC USA

**Keywords:** Physical activity, Schools, Best practices, Policies and programs

## Abstract

**Background:**

The majority of US children do not meet physical activity recommendations. Schools are an important environment for promoting physical activity in children, yet most school districts do not offer enough physical activity opportunities to meet recommendations. This study aimed to identify school districts across the country that demonstrated exemplary efforts to provide students with many physical activity opportunities and to understand the factors that facilitated their programmatic success.

**Methods:**

A total of 59 districts were identified as model districts by members of the Physical Activity and Health Innovation Collaborative, an ad hoc activity associated with the Roundtable on Obesity Solutions at the National Academies of Sciences, Engineering, and Medicine. Semi-structured interviews were conducted with consenting stakeholders from 23 school districts to understand physical education and activity efforts and elucidate factors that led to the success of these districts’ physical activity programming. Districts were geographically and socioeconomically diverse and varied in their administrative and funding structure.

**Results:**

Most districts did not offer the recommended 150 or 225 min of physical activity a week through physical education alone; yet all districts offered a range of programs outside of physical education that provided additional opportunities for students to be physically active. The average number of school-based physical activity programs offered was 5.5, 3.5 and 2.1 for elementary, middle and high schools, respectively. Three overarching and broadly relevant themes were identified that were associated with successfully enhancing physical activity opportunities for students: soliciting and maintaining the support of champions, securing funding and/or tangible support, and fostering bi-directional partnerships between the district and community organizations and programs. Not only were these three themes critical for the development of physical activity opportunities, but they also remained important for the implementation, evaluation and sustainability of programs. These themes also did not differ substantially by the socioeconomic status of districts.

**Conclusions:**

These findings demonstrate the success of school districts across the nation in providing ample opportunities for physical activity despite considerable variability in socioeconomic status and resources. These results can inform future research and provide actionable evidence for school districts to enhance physical activity opportunities to students.

**Electronic supplementary material:**

The online version of this article (10.1186/s12889-018-5889-4) contains supplementary material, which is available to authorized users.

## Background

According to the 2008 *Physical Activity Guidelines for Americans,* children should engage in 60 min of moderate to vigorous physical activity (MVPA) each day and the Institute of Medicine (IOM)^1^, *Educating the Student Body: Taking Physical Activity and Physical Education to School,* advises that more than half of daily MVPA be obtained during the school day [1, 2]. Research suggests that children who meet these requirements have a myriad of health benefits including stronger bones, better cardiovascular health, higher self-esteem, and a decreased risk of chronic diseases such as obesity, hypertension, and diabetes in adulthood [2]. A systematic review by the Centers for Disease Control and Prevention (CDC) also suggests an association between school-time physical activity (PA) and improved academic performance, standardized test scores, and concentration; as well as better attention and classroom behavior [3]. Despite these known benefits, recent evidence reveals that only 42% of children and 8% of adolescents are meeting daily requirements [4]. As such, further research on strategies to increase activity levels and opportunities for children is critical to reduce physical inactivity and the associated health and economic burden.

The *Physical Activity Guidelines for Americans* mid-course report posits schools as an optimal environment for PA intervention [5]. School-based PA interventions have repeatedly been shown to increase activity levels in children and adolescents [6–8] and present an ideal platform for intervention delivery given the time children spend in school and the potential to engage children from diverse socioeconomic backgrounds [9]. The *Physical Activity Guidelines for Americans* mid-course report demonstrates a broad range of school-based interventions as validated mechanisms to increase students’ PA levels, including classroom PA breaks, active transport to and from school, before and after school programs, and physical education (PE). While findings support the effectiveness of these standalone interventions, recent evidence suggests multi-component interventions that combine two or more programs are most successful and provide the greatest benefit to children’s PA levels, fitness, and cognitive ability [5]. Given the multi-disciplinary nature of these interventions, however, additional research is needed to understand the leadership, funding, staffing, evaluation, and support required for implementation.

Despite the known benefits of providing PA opportunities for students, a majority of school districts are not meeting daily recommendations [10]. In 2006, only 3.8% of elementary and 7.9% of middle schools met annual recommendations for PE minutes [11]. In 2012, 58.9% of school districts required and 34.2% recommended that elementary students engage in some amount of daily recess; however, only 30.2% of these districts set targets at or above 30 min [10]. These findings are cause for concern given recent recommendations by the IOM, and demonstrate the need to increase PA opportunities for students during the school day. In addition, while recent research highlights the potential for PA opportunities outside of recess or scheduled PE to increase activity, data reveal fewer than 15% of school districts required PA breaks for elementary and middle school students [12]. Despite this apparent lack of PA opportunities in many school districts, some districts are successfully meeting recommendations and incorporating PA into the school day. However, little formal evaluation has been done with these district programs to identify common strategies for successful implementation. As such, research is needed to understand the programmatic efforts underway in districts meeting PA recommendations to inform strategies in districts that are underperforming.

In 2014, the Physical Activity in Youth Innovation Collaborative (now the Physical Activity and Health) (PA IC) was launched as an ad hoc activity associated with the Roundtable on Obesity Solutions [13] (the Roundtable is an activity within the Health and Medicine Division [formerly the Institute of Medicine] of the National Academies of Sciences, Engineering, and Medicine [the National Academies]). This PA IC is currently comprised of 20 experts in physical activity, exercise and fitness, childhood obesity and health, school, and public policy. Members of the PA IC come from academia, government, non-profit organizations, associations, foundations, and industry. A primary objective of the PA IC was to use existing evidence from successful school- and community-based programs to influence public policies and to map longer-term strategies to increase PA in youth. Therefore, given these interests, this project was conceptualized by members of the PA IC, with the primary objectives to: 1) identify school districts across the country that demonstrated exemplary efforts to provide students with many PA opportunities, 2) understand the factors that facilitated their programmatic success, and 3) develop recommendations for school-based PA interventions and programs that can reach across district demographics and successfully increase PA among America’s youth. Given that this is a novel and under-researched area of study, we utilized a mixed methods approach, which provides more comprehensive data on the many factors that may influence districts ability to provide adequate amounts of PA in their schools. The common, yet unique, strategies identified by this research will fill an important knowledge gap and have the potential for widespread dissemination and impact on the PA and health of America’s youth.

## Methods

This project was conducted by a collaboration between members of the PA IC, who designed the project, established the nomination criteria for identifying exemplary districts, helped with recruiting districts, and provided expert feedback and guidance, and researchers at Tufts University, who implemented the project and carried out the qualitative and quantitative analyses, with some assistance from collaborators at the CDC. Two of the study authors are members of the PA IC, one of whom is also affiliated with Tufts University.

### Nomination criteria

All aspects of the nomination and selection process were carried out by members of the PA IC, an ad hoc activity associated with the Roundtable on Obesity Solutions at the National Academies of Sciences, Engineering, and Medicine. PA IC members identified a convenience sample of school districts in the United States that could best describe exemplary strategies for implementing and sustaining physical activity policies and programs to support students’ PA during the school day. Example exemplary efforts identified by PA IC members included district representative attendance at various physical activity conferences or conventions, partnership with external physical activity organizations, or application for federal funding. PA IC members also identified districts from published write-ups or publicly available data on ongoing physical activity efforts and activity levels among students within the district. The research team aimed to identify a convenience sample of districts that were diverse in geographic location and socioeconomic status (SES). For the nomination process, PA IC members submitted recommended district names to the PA IC Senior Program Officer based on their organizations’ involvement with and knowledge of the ongoing PA activity efforts in each of these districts. The PA IC Senior Program Officer created a master list and removed all duplicate districts. The PA IC discussed and revised the list by consensus, and approved the final nominations over in-person meetings, phone calls, and email. Based on these criteria, PA IC members identified 59 school districts in total.

### Recruitment

Recruitment was conducted by study staff, with assistance from members of the PA IC, from June–October of 2015. Superintendents were contacted via email and asked to identify an individual(s) within the district who could best speak to the PE and PA programs and policies currently underway. Superintendents who did not respond to our initial recruitment emails were followed up with two additional times. When contact information was provided, we subsequently followed up with the individual(s) identified by the superintendent via email and/or phone and asked them to participate in a voluntary phone interview. We attempted to contact these potential interviewees up to three times. If we did not receive a response after the third phone call/email, we stopped recruitment for that district. The interviewees identified by district superintendents assumed various administrative roles within the district including, but not limited to, the: District Director for Health and PE; PE/Health Specialist; Coordinated School Health Administrator; PE Supervisor; Assistant Director of the Whole Student Initiative; and Superintendent.

### Data collection

The interview script was developed by research study staff with input from PA IC members to gain an in-depth understanding of factors that may have contributed to the success of the district’s creation, implementation, and/or long-term maintenance of programmatic and policy efforts around PA, PE, and recess. The survey instrument included questions regarding the reach, initial development, and funding for all programs; the leaders engaged in the development, implementation, and/or continuation of PA programs; the sustainability of programmatic efforts; the implementation and evaluation of programs; equity in program reach; and policy impact. We piloted the initial version of the interview script in March and April 2015. These pilot interviews were conducted with three California districts that differed in size and geographic location to evaluate survey length and comprehensibility. We modified the initial instrument based on feedback from these pilot interviews (See Additional file 1: Final interview instrument).

Between June and October 2015, semi-structured interviews were conducted, via phone, with the final group of recruited districts. For each interview, at least two members of the research study staff were present and took notes on the interviewees responses, as the interviews were not audio recorded. When possible, district characteristics including number of schools, student demographics, and student-to-teacher ratio were collected from the district website to augment the time devoted to non-publicized information. Most interviews spanned 45–60 min depending on the knowledge of the interviewee, the detail provided, and the availability of district data. All protocols and study materials were reviewed by the Tufts University Social, Behavioral, and Educational Research Institutional Review Board. Written consent was not obtained, as data obtained for this study was regarding the school districts and not the individuals interviewed.

Immediately following each interview, research staff reconciled their notes and discussed any inconsistencies and/or points that required further clarification. Wherever necessary, contacts were provided with follow-up questions and clarifications were incorporated into the final dataset. All but one district correspondent responded to these requests for clarification. When clarification was not obtained, this was noted in the dataset. Data were entered into Microsoft Excel and coded. Quantitative data were double entered by two researchers and compared using SAS 9.3 (Cary, NC). Qualitative data were subsequently reviewed for quality by study staff.

### Measures

#### Demographics

District student demographic data on race/ethnicity and SES were recoded based on whether the majority of students (> 50%) were non-white, Hispanic, and/or eligible for free and reduced price lunch (FRPL; y/n). The average number of students served in PRE-K-12th grade, average number of schools, and average student-to-teacher ratio in both PE and academic classes were also calculated and reported. For the purpose of these analyses, district size was determined using the National Center for Education Statistics classification system [14] and modified based on our sample: districts with 1–5 schools were considered small, 6–19 schools were considered midsize, and 20+ schools were considered large. District SES was identified based on the percent of students that qualified for FRPL; districts with 76–100% of students eligible for FRPL were classified as low SES, 26–75% students eligible for FRPL were classified as medium SES, and 0–25% of students eligible for FRPL were classified as high SES [15, 16].

#### Physical activity

Data on minutes of PA/PE provided were recategorized based on whether the district offered at or above IOM recommendations for minutes of school-time PA/PE (150 min/wk. for elementary students and 225 min/wk. for middle/high school students) [1] and/or at or above 20 min/wk. for recess. Minutes of school-based PA could only be assessed based on estimates for PE, since respondents were asked to report on efforts at the district level and few districts had requirements for PA outside of PE classes. District offerings of programs during and after school (school-day PA programs, recess, and after-school PA programs) and district requirements for minutes of recess and/or PE were recorded. The number of different school day PA programs offered (i.e. GoNoodle© and PlayWorks©) were also coded and recorded. Whether districts required PE teacher professional development, hired paid versus volunteer staff for PA efforts, and whether hired staff oversaw PA efforts were calculated and recorded. When available, nationally representative district level data obtained from the CDC’s School Health Policies and Practices Study (SHPPS) were used as a national comparison [10]. The most recent district-level SHPPS data were from 2012. We also evaluated whether there were differences in noted PE requirements reported by interview respondents and archived versions of district websites, wherever available (identified on WayBack Machine: https://archive.org/).

#### Funding

Funding sources utilized by each district were recategorized based on whether they were district/school based, external grants, and/or funding from community organizations or individuals. Internal funding sources were further characterized based on whether they were part of the school department or school level budget, part of the PA budget, part of the district budget, internal grants, part of the state budget, Title I funding or Medicaid funding. We also noted when the districts reported that they required no funding for their PA efforts.

#### Champions

The number and type of champions, defined as an individual or group of individuals who are instrumental in the development, implementation and/or continuation of the PA program(s) in the district, were identified from the following sectors: PE teachers, Principals, Families/Parents, Board of Education members, Parent Teacher Organizations, Superintendents, Community Members/Organizations, Director of Health and PA, Mayor’s Office/County Supervisor/Town Manager, or other.

### Data analysis

Descriptive statistics of quantitative data were conducted in Microsoft Excel and statistical analyses of quantitative data were conducted in RStudio (Version 1.0.153). All qualitative data analyses were carried out using NVivo Version 10. A preliminary codebook was developed based on an initial review of the data and the a priori codes inherent to the interview script. An inductive thematic approach was used for all qualitative analyses to identify explicit and implicit themes within the data [17]. Six districts that represented the demographic diversity of the total interview sample were randomly selected and independently coded by two study staff. After independently coding the first two interviews, staff met to discuss new themes, any necessary adjustments in the existing codes, and any discrepancies in coding. This process was repeated in NVivo until the two researchers established inter-rater reliability (≥ 80% agreement) on all codes. Emergent themes were identified by study staff, discussed with members of the PA IC, and further developed based on feedback from PA IC members. Ultimately, three overarching factors were identified as integral to providing many PA opportunities to students: having champions, funding and support, and bi-directional partnerships with the community and external PA programs. Themes were evaluated overall and by district SES; results are presented below wherever appropriate. We were not able to discuss differences in our findings based on geographic location, as there was only one rural district and at least three participants are required in each group to evaluate qualitative differences [18]. Quotes presented below were modified to de-identify participating districts throughout.

## Results

### Sample

A total of 59 school districts from all three socioeconomic sectors (29% low; 59% middle; 12% high) and geographic locations (53% urban; 32% suburban; 15% rural) were identified through the nomination process, and contacted. Of these districts, 1 declined to participate, 35 did not respond to multiple requests to participate, and 23 were successfully recruited and interviewed. No significant differences were observed between participating and non-participating school districts in geographic location or socio-economic status (Additional file 2: Table S1).

Demographics and characteristics of the participating school districts are presented in Table 1. Participating districts were primarily urban (56%), middle SES (48%), and large (82%); with an average of 155 schools and 115,695 enrolled students from PRE-K – 12th grade. Based on enrollment, 48% of participating districts were among the 100 largest US school districts during the 2012 fiscal year [19]. The average student-to-teacher ratio in PE classes was higher than that of academic classes, with the largest in elementary schools, comparatively. More than half of the districts had a majority non-white and non-Hispanic student population; and in 83% of districts, a majority of students were eligible for free and reduced priced lunch (average, 63%).

**Table 1:** Characteristics of the participating school districts (*n* = 23)

*District demographic and size characteristics*	
Geographic location (%)	
Urban	56
Suburban	39
Rural	4
Socio-economic status (%)	
Low SES	39
Medium SES	48
High SES	13
District enrollment PRE K – 12 (mean, range)^a^	115,695 (~ 2100 – ~ 650,000)
District size (%)^b^	
Small	9
Midsize	9
Large	82
Student-to-teacher ratio, academic class (mean, range)	16.31 (10.55–23.55)
Student-to-teacher ratio, PE class (mean, range)	
Elementary	53.4 (17–60)
Middle	48.7 (25–60)
High	51.0 (15–60)
*Student socio-demographics*	
Race (%)	
Majority non-white	52
Ethnicity (%)	
Majority Hispanic	22
Majority students eligible for free/reduced lunch (%)	83

^a^The district enrollment range values provided were rounded down to de-identify participating districts

^b^For the purpose of these analyses, districts size measure were determined using the National Center on Education Statistics classification [14] and modified based on our sample: districts with 1–5 schools were considered small; 6–19 schools were considered midsize and 20+ schools were considered large

### Overview of students’ physical activity levels and district PE/PA offerings

Table 2 presents an overview of the PE and PA efforts underway in participating districts and, whenever data were available, we provide a comparison to SHPPS 2012 data [10]. Overall, weekly PE requirements varied by school level (elementary/middle/high): having a weekly PE requirement was a more common practice in elementary and middle schools. Districts in our sample were more likely to have a weekly PE requirement compared to a national sample. The majority of respondents provided estimates around PE requirements that were consistent with what was noted on archived versions of district websites (92% of responses matched what was listed on the district webpage).

**Table 2:** Physical education and physical activity program characteristics in elementary, middle, and high schools in the participating school districts (*n* = 23)

	Elementary	Middle	High
*PE requirements and offerings, overall and in relation to recommendations*			
Sample districts with a minimum PE requirement (min/wk) for students (%)	87	87	78
Nationally representative districts with a minimum PE requirement for students (%)^a,b^	78	72	80
Sample district PE requirement, min/wk. (mean, range)	96 (0–225)	160 (0–275)	184 (0–337)
District PE requirement meets or exceeds IOM rec^c^ (%)	17	35	52
*PA offerings overall*			
Sample districts that require recess for students (%)	74	22	0
Nationally representative districts that require recess for students (%)^a^	59	–	–
Sample districts that offer school-based PA programs (%)^c^	100	74	61
Nationally representative districts that offer school-based PA programs (%)^a,d^	45	34	14
Average number of school-based PA programs offered in sample districts (mean, range)	5.5 (2–14)	3.5 (0–10)	2.1 (0–7)
Average number of after-school programs offered in sample districts (mean, range)^e^	1.5 (0–3)	1.7 (0–3)	1.1 (0–3)

*Abbreviations*: *PE* Physical education; *min* Minutes; *wk* Week; *IOM* Institute of Medicine; *rec* Recommendations

^a^Nationally representative data were presented wherever available. Results from the nationally representative sample of districts were abstracted and calculated from the Centers for Disease Control’s School Health Policies and Practices Study (SHPPS) [10]. The most recently available data on Physical Activity and Physical Education were from 2012

^b^Respondents were asked to indicate whether their district had any time requirements (min/wk., credit hours/year, etc.), so we are unable to differentiate based on min/wk. alone

^c^IOM recommends 150+ min/wk. of physical activity for Elementary students and 225+ min/wk. for middle/high school students. These values only represent time spent in PE and do not account for time spent in other sources of physical activity (i.e. recess, classroom PA breaks, etc.)

^d^School day PA programs defined as any program offered during the school day outside of PE (includes classroom physical activity breaks)

**Fig. 1:**
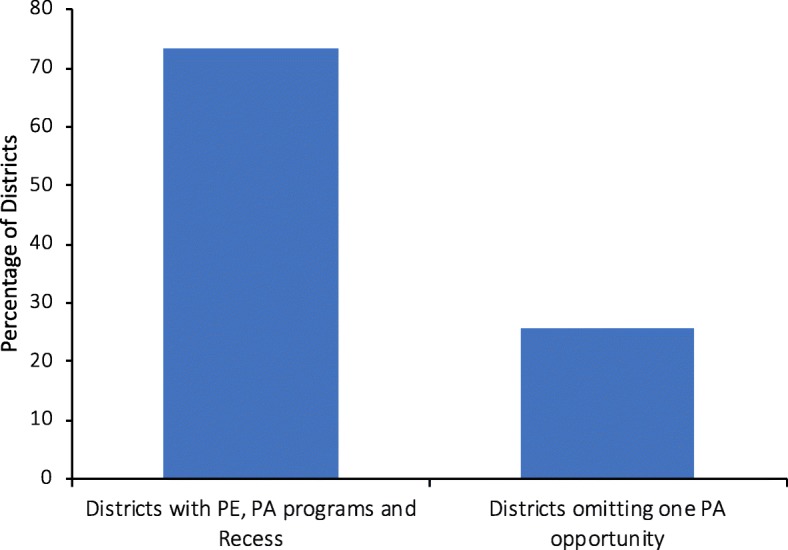
The percentage of districts that implement multiple physical activity opportunities during the school day including PE, school time physical activity programs outside of physical education, and recess. At least two or more opportunities were available in all districts. Results presented here are across all school levels (Elementary, Middle, High). Abbreviations: *PE* Physical education and *PA* Physical activity

Most district respondents estimated students received between 60 and 150 min or more of PE per week at their Elementary schools, between 45 and 225 min or more of PE per week at their Middle schools, and between 135 and 225 min or more of PE per week at their High schools (though many districts only required PE a limited number of credits for middle and high school students, so these responses represent their estimations for when students were actually enrolled in PE classes). Although a majority of districts estimated that students received enough PE to meet their district PA requirement (74% elementary; 78% middle; 70% high), a relatively smaller percentage of districts estimated that students met or exceeded IOM recommendations for minutes of school time PA [2] through weekly minutes of PE. Additional practices used to augment PE offerings included providing recess and implementing other school-based PA programs (e.g. class-time PA breaks, during-school walking/running programs) and after-school programs. Such practices were employed in 100% of participating districts, with 74% implementing a combination of all three (recess, school-based and after-school PA offered outside of PE) (Fig. 1). Furthermore, the provision of recess and classroom PA breaks were more common practices in participating districts compared to the national, with considerable variation by school level (Table 2).

**Fig. 2:**
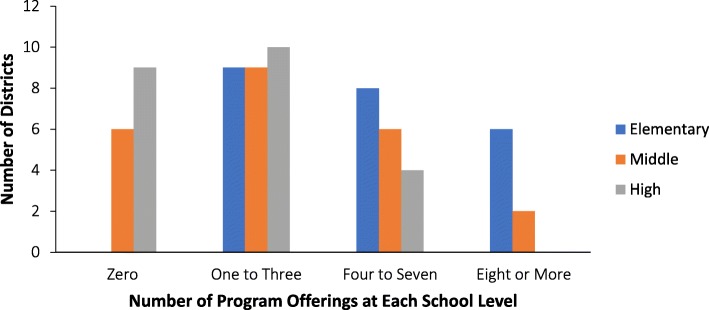
The number of districts with a range of program offerings at each school level (elementary, middle, and high). The number of programs at each level was identified for each district and then divided into ranges so as to show the number of districts that offer some programs versus many

PA programs offered during the school day were provided in the majority of districts at the elementary, middle, and high school level (Table 2); and non-interscholastic after-school programs were also offered in 91% of district’s elementary schools, 95% of the district’s middle schools, and 75% of the district’s high schools. Furthermore, many districts offered a variety of different programs, with the majority of districts offering 6–10 programs across their elementary, middle, and high schools (data not shown); most programs were offered in elementary schools compared to middle or high schools (Fig. 2). On average, low SES districts implemented more PA programs compared to the middle and high SES districts (13 vs. 8 vs. 9, respectively). Recess was also offered in about three quarters of the districts at the elementary school level, and in about a fifth of districts at the middle school level (Table 2).

**Fig. 3:**
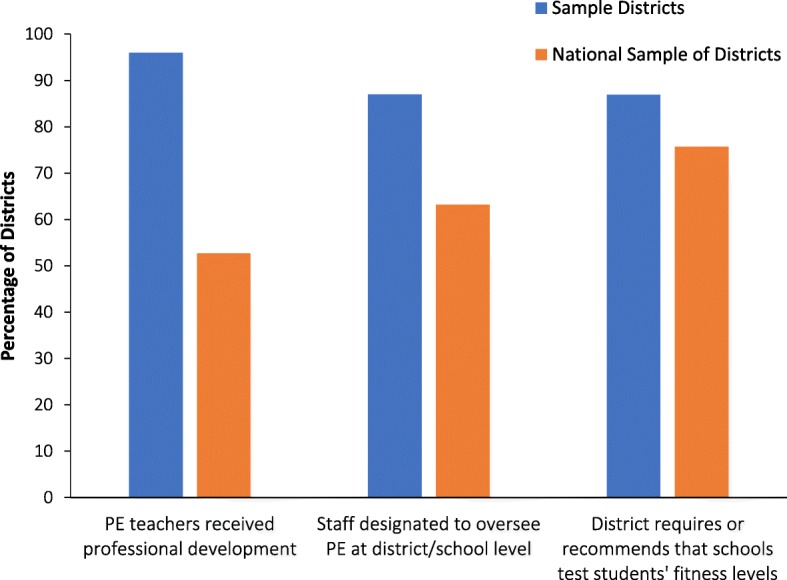
The percent of districts that require professional development for PE teachers, have hired staff to oversee PE efforts, and/or require or recommend that schools test students’ fitness levels. The data on the national sample of districts used as a comparison were abstracted from the Centers for Disease Control’s School Health Policies and Practices Study (SHPSS) 2012 report [10]. Abbreviations: *PE* Physical education

PE and PA staff support were integral to program implementation. The presence of staffing designated to overseeing PE efforts was more common in the participant (87%) than national (63%) sample (Fig. 3). Additionally, a higher proportion of PE teachers and staff received some form of professional development from participating districts, compared to those in the national sample and a higher proportion of districts in our sample evaluated student fitness levels compared to districts in the national sample (Fig. 3).

### Overview of key factors facilitating school-based physical activity in exemplary districts

Three factors were identified as thematically important to providing students with many PA opportunities: (i) champions; (ii) funding and tangible support; and (iii) bi-directional partnerships with the community and external PA programs. According to respondents, these factors were considered integral to the creation, implementation, and sustainability of district-wide PA programmatic efforts.

#### Champions

**Fig. 4:**
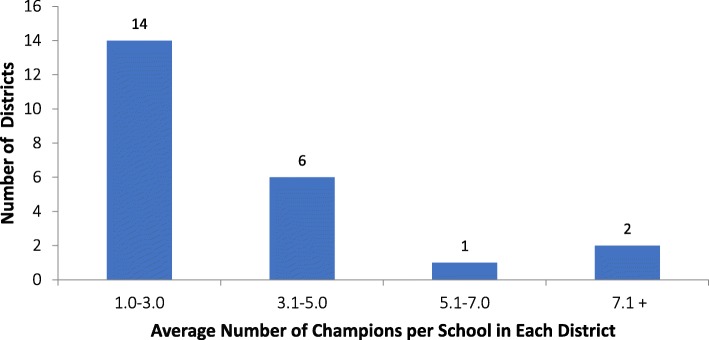
The average number of champions (across all categories or types—PE teachers, principals, families, parent teacher organizations, etc.) per school in each district. The average number of champions per school was calculated by dividing the total number of champions by the total number of schools

Champions were central to the PA efforts of all districts, regardless of district SES. In 87% of districts there were established staff positions for the oversight of PE/PA programming, and these staff often served as champions—developing and advocating for district PA efforts. All districts identified 9 or more champions in total and the majority included the involvement of champions from more than 9 of the following sectors: PE teachers, Principals, Families, School Board, Superintendent, Community Members, Parent Teacher Organization or Parent Teacher Association, the Director of Health and PA, Mayor, and Other (responses included Sports Teams, Universities, local Departments of Public Health, etc.). On average, districts reported the involvement of approximately 3 champions per school within their district across all PE and PA efforts, with the majority of districts reporting 1–3 champions per school (Fig. 4).

#### Funding and tangible support

**Fig. 5:**
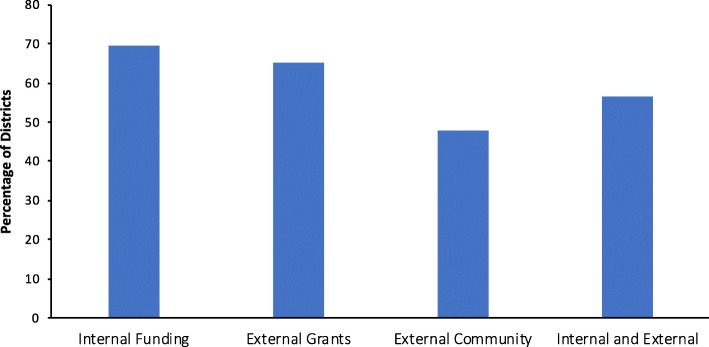
The percent of districts that utilize internal funding, external grants, community organization funds (external community), and/or internal and external sources of funding are presented here. External community funding sources were organizations such as hospitals, local business, etc. that provided funding for district programs

All respondents reported receiving some type of funding and/or tangible support (data not shown). Many districts received support by way of traditional methods, such as grants (i.e. Carol M. White Physical Education Program (PEP) and Community Transformation Grants), fundraisers, district budgets, and governmental support (CDC, Parks and Recreation staff, U.S. Department of Education, State Department of Education; Fig. 5). Nine percent of districts obtained support from external organizations, allowing their programs to operate at low or no cost, while 39% of districts procured funding from more creative sources, including private donations (from parents, foundations, or businesses) and/or support from local organizations and businesses (hospitals, local Zumba instructors, First Tee, and Universities). Over half of the districts (57%) utilized a diverse portfolio of funding sources, including both internal (district or school budget) and external sources (community donations or grant funding). Funding and tangible support were devoted to both start up and ongoing program needs, including: staff salaries, equipment, professional development, building new centers, program software and/or program development. External organizations also provided support by way of resources for PA programming, such as equipment, work out facilities, curriculum and teacher training, and expertise.

#### Bi-directional partnerships

Most district respondents mentioned partnerships with community organizations and/or national PA programs as key avenues to augment the resources and opportunities for PA programming at schools. Reportedly, partnerships further promoted the provision of funding/tangible support and support from champions, highlighting the importance of these two factors to school PA programming. Partnerships between districts and the community organizations were of a “bi-directional” nature, originating from both the organization (i.e. program officials reaching out to districts to offer free trials or equipment, followed by long-term cooperation) and the district (i.e. school staff contacting program officials, or attending conferences run by programs). These relationships were sustained through cooperation of both stakeholders. The types of partnerships varied from local hospitals and businesses to nationally recognized programs such as GoNoodle© and PlayWorks©. These partnerships further supported school PA programs by way of equipment donations, the availability of workout facilities, resources for evaluation metrics, teacher training and curriculum, expertise, and program instructors.

### Putting evidence into practice: How champions, funding and tangible support, and bi-directional partnerships contributed to the program “life-cycle”

The following section details how champions, funding and tangible support, and bi-directional partnerships contribute to program creation, implementation, and evaluation for the nominated districts.

#### Program ideation

The majority of districts developed programs internally (78%) using a bottom-up/grassroots approach (developed at the school level by students, school staff etc.; 17%), a top-down approach (developed by leadership/staff at the district level such as a district director of health and education, a district health board etc.; 39%), or a combination of the two approaches (22%). When districts provided external programs it was often in response to the receipt of grant funding (i.e. SPARK) and/or external PA program support (e.g. GoNoodle© and PlayWorks©) by way of organizational funding and bidirectional partnerships.

Supportive leadership from within the district was integral to program ideation, particularly for low SES districts. Champions played a fundamental role in program development and were most often involved in grant writing, policy formation and development, coordinating and advocating for efforts across organizational levels, fostering relationships with community partners (or other community champions), and fundraising. Several respondents commented on the importance of district leadership buy-in to building and maintaining enthusiasm for PA programs:

“*A lot of ideas came from [the District Director’s] office. PA breaks gained traction by educating principals, community support, and advocacy from his office” –D8*

*“The biggest barrier is shifting the culture and mindset of the staff; an enthusiastic superintendent helped to overcome this barrier”—D24*

Bi-directional partnerships and funding/tangible support were also integral to program ideation. PA program representatives reached out to personnel within the district to offer either equipment or a trial of their program, and additional program ideas also came from external champions including local politicians, PA program representatives, and conferences. District representatives also attended conferences where they brought back ideas to the schools in their districts for future programming.

*“The mayor started an initiative to lengthen the school day in an effort to increase opportunities for physical activity, including incorporating recess into the lunch block. The mayor also engaged community partners and parents.”—D15*

*“The programs were grassroots efforts and included brainstorming with community partners and organizations and developing ideas. They focused on how to incorporate physical activity into the classroom easily and in a sustainable way.”-D16*

External funding also informed program development, but this was cited less often (funding and tangible support):

*“The community transformation grant brought professional development training and PE curriculum development, the district also received a partnership to improve community health grant (CDC) that provided professional development for teachers.”—D19*

#### Program implementation

Once the PA programs were created, champions and bi-directional partnerships facilitated program implementation via support or advocacy from the district respondent and external organizations. In addition, staff and volunteers were involved in the PA efforts of the majority of school districts (65% of the 20 districts that provided information on staffing; three districts did not have data on staffing), with paid staff primarily being utilized during the school day and volunteer staff in out-of-school time programs. Four districts used only paid staff and no districts reported using solely volunteer staff. While specific roles of involved paid staff were not identified during interviews, many programs took place outside of PE and were facilitated by classroom teachers, suggesting that classroom teachers also served as champions of PA programs.

Strategies did not differ substantially by SES, but more champions per school were involved in the programmatic efforts of high SES districts relative to middle and low SES districts (5 vs. 2 vs. 3 champions per school, respectively).

*“Teachers facilitate and support physical activity after school, with clubs, running and walking. The Health and Wellness Division is working with central administration and teachers to develop a plan for physical activity promotion. It is important to emphasize the value of supportive leadership. Programs are built over time with a strong level of interest in physical activity by the superintendent and school support staff. They have dedicated staff members to champion physical activity efforts.”—D11*

For many districts, funding and tangible support were key factors in program implementation. Overall, start-up costs were higher than ongoing costs, and the biggest barriers to obtaining start-up funding were budget cuts or small budgets, difficulties in obtaining grant money, and competing educational priorities within the district (academic vs. health). Many districts were creative in dealing with funding challenges, seeking low or no cost programs and/or identifying innovative solutions to budget constraints. External programs also provided training and free resources/equipment, highlighting the importance of bi-directional partnerships for program implementation.

*“School district budget cuts are the biggest challenge. To overcome this barrier, they form partnerships with outside organizations that provide funding and equipment for PA programming, such as the USTA (tennis racquets), First Tee (golf curriculum and resources), the children's hospital (donations every year for incentives, registration fees for events), and a local bowling alley (rotated through schools 2 weeks at a time).”—D4*

Though funding was important for program implementation for all districts, funding challenges differed by district SES. For example, high SES districts did not always qualify for grant funding, so they needed to procure funding from other sources, yet these districts almost always had internal, district funds that were allocated towards PE/PA programming*.* Low SES districts were less likely to cite examples of external organizations/individuals as champions than respondents from middle and high SES districts.

#### Program sustainability and evaluation

Champions were repeatedly cited as being integral to the long-term continuation of programmatic efforts. Factors that were attributed to being positively associated with the sustainability of PA efforts include: the respondent’s “continued efforts in professional development and funding” and having a supportive Board of Education/Superintendent. District respondents were also responsible for ongoing evaluation of programs, providing evidence of the success of their programs, and advocating for their district’s efforts. Many champions aimed to create a culture of health and wellness in their districts, noting the importance of a culture shift to support long-term sustainability.

Several district respondents also voiced the importance of reporting program outcomes and results from the scientific literature for the long-term sustainability of the program. Almost all school districts used some sort of implementation (91%) and outcome evaluation (87%) method. Implementation evaluation methods varied and included staff observations/evaluations, tracking use within software programs (i.e. for GoNoodle), and teachers/staff self-reporting their use of the program(s). Urban and suburban districts reported more of a need for implementation evaluation to report to granting agencies compared to the rural districts. Outcome evaluation methods were more standardized across districts, with most schools implementing FitnessGram (13/20), other fitness tests or self-reported PA (10/20), or anthropometric measures (4/20):

*“….. providing ‘the evidence’: when a program is successful, then trickles up to decision makers (good programs lead to good results, which get publicized), communication of and recognition for what works, showing positive outcomes (data shows the value of a program and validates efforts)”* –*D12*

*“The district had to ‘cut a significant amount from the budget, but they did not cut any PA program [funding] because of the strength of the program”.--D2*

Funding was commonly cited as a barrier to sustainability and district respondents highlighted the creativity of administrators/staff as imperative to continued PA efforts:

*“Funding is required, but [the district] look[s] at innovative ways to sustain the program without incurring a high cost, such as the use of Parks and Rec staff, who are funded through the county. They have gap periods during which they help with PA programs in the schools, so this eliminates the need for schools to pay staff (resourceful).” – D15*

Although champions, the availability of funding, and/or continued support from external organizations were cited as being important for the sustainability of programmatic efforts, variation was noted across SES groups. When asked to identify factors that were positively related to the sustainability of PA efforts, respondents from low SES districts were more likely to cite factors at the leadership/policy level, such as state mandates and the support of the Superintendent, whereas respondents from middle SES districts were more likely to reference factors at the school level, such as teacher buy-in. Evaluation efforts were prioritized across all SES levels, but respondents from high SES districts were more likely to cite the importance of research and evaluation data to the sustainability of their programs. Low SES districts were more likely to cite competing priorities for teachers as a factor that negatively impacts the sustainability of PA efforts, whereas middle SES districts were more likely to cite lack of “teacher buy-in” as potentially being a barrier to the long-term success of PA efforts.

## Discussion

Despite variability between the sample districts based on district size, the availability of resources, and SES, the results of this project demonstrate that school districts can provide substantive PA opportunities to students to increase the likelihood that 30 min of PA is acquired during the school day. We identified three overarching and broadly relevant themes that were associated with these districts’ successful efforts to enhance PA opportunities for students: champions, funding and tangible support, and bi-directional partnerships. Not only were the three themes key correlates of the development of PA opportunities for students, but each theme also remained important for the implementation, evaluation, and sustainability of programs. Furthermore, each theme supported the development of a culture of health within the schools and districts, which was frequently cited as a contributing factor to the success of enhancing PA opportunities for students.

A culture of health, which can be broadly defined as an environment that supports good health and well-being, was often associated with having a superintendent or upper level leadership member who identified PA for students as a district priority. In our sample, respondents who identified the superintendent as a champion consistently emphasized the importance of this upper level leadership in the success of their programs. Many of these respondents also mentioned that administrations who were supportive of PA often developed a funded position for a lead champion for PA programmatic efforts; and these lead champions were frequently involved in acquiring funding and tangible support for district programs, as well as the developing and maintaining bi-directional partnerships. As such, the synergistic nature of the three identified themes may be enhanced by district leadership that prioritizes PA for students and supports a lead PA champion.

In 2014, the CDC and SHAPE America released a guide for developing a Comprehensive School Physical Activity Program (CSPAP), which is a multi-component model for school districts and schools to enhance opportunities for students to be physically active. The model suggests five important components that contribute to PA opportunities for students: PE, PA before and after school, PA during school, staff involvement, and family and community engagement [11]. While there is a lack of data to suggest whether schools and districts meeting PA recommendations are following the CSPAP guide, the efforts identified by respondents in this sample closely align with the CSPAP, with the majority of districts (74%) providing PA during school, before or after school, and PE, as well as noting staff involvement and champions at the family and community levels. The inclusion of champions at the staff, family and community levels, as recommended by the CSPAP model, supports the development and sustainability of bi-directional partnerships and funding and tangible support.

While there were limited differences in results across district SES, suggesting the potential for widespread and meaningful impact, this project was not met without limitations. First, the study design did not allow us to draw any conclusions on causality or to directly attribute the success of the included to any one of the identified factors. We also cannot determine whether the PA programs identified here were responsible for the success of these districts, if it is a combination of the identified factors, or if it is the result of unmeasured factors. However, given the limited prior work in this space, we aimed to generate hypotheses about how these factors interact to facilitate successful PA programming. Future controlled studies should evaluate the combined impacts of the factors uncovered here on student PA levels. Second, the school districts interviewed were identified by way of convenience sampling and agreed upon by group consensus through the nomination process. Some members of the PA IC were involved with youth PA funding agencies, which may have led to the identification of districts that have received funding, introducing the potential for bias among our results. Given the response rate, it is also possible that those districts that decided to participate were among those providing the most physical activity opportunities for their students, compared to those that did not respond. Third, interview data were not recorded and thus not transcribed verbatim. While this could have introduced the potential for missing data, having two research staff present during each interview mitigated this possibility. In addition, our sample did not include underperforming districts as a comparison group; however, we were able to compare our sample against the SHPPS data for national comparison. Although these data were collected three years prior to the start of this project, comparison of reports collected in 2006 [12], 2012 [10], and 2016 [20] show a high degree of similarity at the district-level suggesting that these estimates may not have changed substantially over time (See Additional file 2: Table S2).

We also acknowledge the possibility of reporting bias with regard to weekly physical activity levels. It’s possible that social desirability biases lead respondents to over-report weekly minutes of physical activity, given their awareness of the purposes and underlying goals of this study. Additionally, given the variability in how districts reported their requirements, it’s also possible the varied timeframes (e.g., semester requirements versus requirements spanning the full school-year) might have led to over-reporting since we did not adjust for time in our analyses. Yet, in this sample of school districts, variability in PE requirements was reported almost exclusively at the high school level, and would likely not have impacted estimates at the lower grade levels. Moreover, in reviewing PE requirements listed on archived versions of district websites, we found that the respondents’ estimates were consistent with those listed on the district site, and many of these districts offered enough PE to meet these requirements. Alternatively, while interviewees were identified by superintendents as individuals most knowledgeable of PA programmatic efforts, district level contacts may not have been fully aware of all efforts taking place at the school level, which may have led to underreported PA efforts. We also have evidence that students likely engaged in additional physical activities outside of district-PE requirements, which would not have been captured in our analyses and would contribute an additional source of under-reporting. Despite identified limitations, this project fills an important research gap, provides a foundation for future research, and presents evidence to inform school district’s efforts to enhance PA opportunities to students nationwide.

## Conclusions

The findings presented here are encouraging—districts across SES groups and geographic locations have demonstrated the ability to successfully offer a myriad of PA opportunities for their students, outperforming a nationally representative sample of districts. Their success is associated with three synergistic methods: (i) soliciting and maintaining the support of champions, (ii) securing funding and/or tangible support, and (iii) fostering bi-directional partnerships between the district and community organizations and programs. These best practices appear to be not only critical to the development and implementation of these districts’ PA programs, but also to the support of their ongoing success. The districts in this study have made commendable efforts to ensure that all students receive ample opportunities to be physically active throughout the school day and have provided a road map of best practices that can be utilized by other districts across the country to help promote the wellbeing of school-aged children in the United States.

## Additional files

Additional file 1: Final interview instrument. (DOCX 49 kb)

Additional file 2: **Table S1.** Demographic characteristics of participating versus non-participating districts (*n* = 59)^a,b^. **Table S2.** Comparison of physical education and physical activity program characteristics of nationally representative samples from the SHPPS 2006, 2012, and 2016 reports. (DOCX 16 kb)

## Abbreviations

CDC: Centers for disease control and prevention
CSPAP: Comprehensive school physical activity program
FRPL: Free and reduced-price lunch
IOM: Institute of Medicine
MVPA: Moderate to vigorous physical activity
PA IC: Physical Activity in Youth Innovation Collaborative
PA: Physical activity
PE: Physical education
PEP: Physical Education Program
PRE-K: Pre-kindergarten
SES: Socioeconomic status
SHPPS: School Health Policies and Practices Study

## Footnotes

1 As of March 2016, the Health and Medicine Division of the National Academies of Sciences, Engineering, and Medicine continues the consensus studies and convening activities previously undertaken by the Institute of Medicine (IOM).
