# Supplementary material for: Investigating best practices of district-wide physical activity programmatic efforts in US schools– a mixed-methods approach
Source: BMC Public Health. 2018 Aug 30;18:992. doi: 10.1186/s12889-018-5889-4 (PMC6117892; doi:10.1186/s12889-018-5889-4)
Supplement: Supplementary file 1 — Final interview instrument. (DOCX 49 kb) [file 12889_2018_5889_MOESM1_ESM.docx]

**APPENDIX A**

**District Contact Phone Interview Script**

**(Interview should take ~30 minutes)**

**Date of interview:** ____________________

**Interviewer name:** ________________

**District name:** ___________________

**Interviewee name:**__________________

**Interviewer script:** Hello. This is ___________________________calling on behalf of Tufts University. Thank you for taking the time to talk with us today. This voluntary interview will focus on the programmatic efforts around physical activity in your school district. This project was initially developed as a part of the Institute of Medicine’s (IOM) Innovation Collaborative on Physical Activity (PA IC) and is being evaluated by PA IC members at Tufts University. Your district was identified by Tufts University and the IOM PAIC and as being exemplar in your efforts to increase physical activity opportunities for your students.

The entire interview should only take approximately 30 minutes of your time and your participation is greatly appreciated. We will use the information from these interviews to summarize what makes up successful physical activity programs and what it takes to implement these programs. The information gathered from these interviews will be used in reports, presentations and publications. We also aim to translate our findings into advocacy and policy efforts.

All of your comments today will be kept confidential. Your name will not be used in any of the publications or materials resulting from these interviews. However, with your permission, your district’s name may be used in materials resulting from these interviews **[Circle the response: The respondent approves/does not approve of using the district’s name]**. We will follow up with you and your superintendent via email to confirm that it is permissible to use the district’s name. The district’s name will not be used unless we receive electronic confirmation from both you and the superintendent.

You may choose not to complete the interview, may stop the interview at any time, and may choose to not answer specific questions. __________________________ will be taking notes during our interview today. If you’d like any part of our conversation today to not be notated, please let me know and we will not write it down. Do you have any questions before we begin?

1. **Overall District Characteristics [Where possible, pre-enter information from online databases and confirm data with the interviewee]:** First, we’d like to get more information about the overall characteristics of your district including information on where your district is located, the make up of your student body, and the physical activity programs in your district.
   1. **[If data is available, skip to 1b]** Is your district urban, suburban, or rural **[circle one]**? ……………………………………………………………………………………………Urban Suburban Rural
   2. **[If data is available, skip to 1c]** How many enrolled K-12 students do you currently have? ........................................................................................................................................._______
   3. **[If data is available, skip to1d]** Do you have pre-K? If, so how many students? ..........................................................................................................................................._______
   4. **[If data is available, skip to 1e]** What is the average student to teacher ratio in your district for academic classes?................................................................................................................._____
   5. **[If data is available, skip to 1f]** What is the average student to teacher ratio in your district for **physical education** classes in your **[Elementary, Middle, High schools]?**

| **Elementary schools** | **Middle schools** | **High schools** |
| --- | --- | --- |
| __________________ | __________________ | __________________ |

- 1. Demographics:
     1. **[If data is available, skip to 1fii]** What is the racial/ethnic composition of the students in your district?
        1. Race
           1. % Caucasian…………………………………………………………………….______
           2. % Black or African American……………………………..……………._______
           3. % American Indian or Alaskan Native………..……..……………._______
           4. % Asian………………………………………..……..………………….………._______
           5. % Native Hawaiian and Pacific Islander………..…..…...………._______
           6. % Other race……………………………………………………………………_______
           7. % Two or more races…………………………..………..…..…...…..…._______
        2. Ethnicity
           1. % Hispanic or Latino…………….…………………………………………._______
           2. % Not Hispanic or Latino.……………………………..……………..…._______
     2. **[If data is available, skip to 1g]** What percent of the students in your district qualify for free and reduced lunch? …………………………………………………………………………………………………………… ____________

|  | **Elementary schools** | **Middle schools** | **High schools** |
| --- | --- | --- | --- |
| **g. [If data is available, skip to 1h]** How many **[Elementary, Middle, High]** schools are there in your district? | __________ | __________ | _________ |

|  | | **Elementary schools** | | **Middle schools** | | **High schools** | |  |  |
| --- | --- | --- | --- | --- | --- | --- | --- | --- | --- |
| **h.** How many Physical Education teachers are funded or on staff in your district’s **[Elementary, Middle, High]** schools? | | __________________ | | __________________ | | __________________ | |  |  |
|  | | | **Elementary schools** | | **Middle schools** | | **High schools** | | |
| **i. [If data is available, skip to 1j]** During the past two years, did any physical education teachers or specialists at your district receive professional development (e.g. workshops, conferences, continuing education or any kind of in-service) on physical education **[circle one]**? | | | **No**  **Yes** | | **No**  **Yes** | | **No**  **Yes** | | |
|  | | **Elementary schools** | | **Middle schools** | | **High schools** | | |  |
| **j.** How many minutes of physical education are required in your **[Elementary, Middle, High]** schools per week? | | _____________sessions/wk  ____________min/session  ___________wks of delivery | | _____________sessions/wk  ____________min/session  __________wks of delivery | | _____________sessions/wk  _____________min/session  ___________wks of delivery | | |  |
|  | | **Elementary schools** | | **Middle schools** | | **High schools** | | |  |
| **k.** How many minutes of physical education do students in **[Elementary, Middle, High]** school get per week **[circle one]**? | | ___0-29___ min/wk  __30-59___ min/wk  __60-89___ min/wk  __90-119___ min/wk  _120-149___ min/wk  _150 or more min/wk | | ___0-44____ min/wk  ___45-89___ min/wk  ___90-134___ min/wk  __135-179___ min/wk  __180-224___ min/wk  225 or more min/wk__ | | ___0-44____ min/wk  ___45-89___ min/wk  ___90-134___ min/wk  __135-179___ min/wk  __180-224___ min/wk  225 or more min/wk__ | | |  |

|  | **Elementary schools** | **Middle schools** | **High schools** |
| --- | --- | --- | --- |
| **l**. What percentage of students in your **[Elementary, Middle, High]** schools participate in weekly physical education classes **[circle one]**? | **1.** Less than or around one quarter of students  **2.** Between one quarter and a half of students  **3.** Between one half and three quarters of students  **4.** Between three quarters and all students | **1.** Less than or around one quarter of students  **2.** Between one quarter and a half of students  **3.** Between one half and three quarters of students  **4.** Between three quarters and all students | **1.** Less than or around one quarter of students  **2.** Between one quarter and a half of students  **3.** Between one half and three quarters of students  **4.** Between three quarters and all students |
|  | **Elementary schools** | **Middle schools** | **High schools** |
| **m.** Is recess or **active free time** required in [**Elementary, Middle, High]** school **[circle one; If no continue to question 1.n. If yes, continue to 1.m.i.]**?  **mi.** If yes, approximately how many minutes per week for recess **[circle one]**? | No  Yes  ______N/A______  __1-9___ min/wk  __10-19___ min/wk  _20 or more min/wk_ | No  Yes  ______N/A______  __1-9___ min/wk  __10-19___ min/wk  _20 or more min/wk_ | No  Yes  ______N/A______  __1-9___ min/wk  __10-19___ min/wk  _20 or more min/wk_ |
|  | **Elementary schools** | **Middle schools** | **High schools** |
| **n.** How many after school (club, non-interscholastic) physical activity programs do your **[Elementary, Middle, High]** schools conduct per week **[circle one]**? | _______0_________  _______1 - 3_______  ________4-6_______  ______7 or more____ | _______0_________  _______1 - 3_______  ________4-6_______  ______7 or more____ | _______0_________  _______1 - 3_______  ________4-6_______  ______7 or more_____ |

1. **Reach for PA programs:** Now, we’d like to ask you about the physical activity programs in your district that occur during the school day.

|  | **Elementary schools** | **Middle schools** | **High schools** |
| --- | --- | --- | --- |
| **a.** **[If data is available and response is yes, skip to 2.a.i. If data is available and answer is no, skip to 3.]** Outside of physical education, do students participate in physical activity breaks in classrooms during the school day at your district’s **[Elementary, Middle, High]** schools **[If no, continue to question 3. If yes, continue to 2ai. and 2aii.]]**?  **ai.** Including classroom activity breaks, which physical activity programs are you currently implementing in your district’s **[Elementary, Middle, High]** schools? When answering, please indicate if school participation is voluntary or mandatory.  aii. How many years have each of these programs been in place?  **aiii**. Are any of the physical activity programs outside of your Physical Education classes a part of a district-wide policy? If so, how many program(s)? | **Yes**  **No**  ___________________  ___________________  _________________________________________________________  ___________________  ___________________  ___________________  _________________________________________________________  ___________________  ___________________ | **Yes**  **No**  _____________________________________________________________________________________________________  __________________________________  ___________________________________________________  _________________  _________________ | **Yes**  **No**  _________________  _____________________________________________________________________________________  _________________  __________________________________  ___________________________________________________  _________________ |
|  | **Elementary schools** | **Middle schools** | **High schools** |
| **b.** How many schools are currently participating in the physical activity program(s) at the district’s **[Elementary, Middle, High]** schools? | __________________ | _________________ | _________________ |
| **bi.** At **[Elementary, Middle, High]** schools with programs, approximately what percent of teachers actually implement these programs? | __________________ | _________________ | _________________ |

1. **Reach and Initial Program Development and Funding for all programs:** The following questions review the reach, initial development and funding efforts for the physical activity programmatic efforts in your district including any of the physical activity programs mentioned earlier in the interview, physical education classes, classroom physical activity breaks, before school physical activity programs, active recess, etc.

|  | **Elementary schools** | **Middle schools** | **High schools** |
| --- | --- | --- | --- |
| Thinking about **all of the activities** that the district has around physical activity in schools, what is the approximate proportion of **children** that these programs/activities reach within **[Elementary, Middle, High]** schools implementing the program(s) **[circle one]**? | **1.** Less than or around one quarter of students  **2.** Between one quarter and a half of students  **3.** Between one half and three quarters of students  **4.** Between three quarters and all students | **1.** Less than or around one quarter of students  **2.** Between one quarter and one half of students  **3.** Between one half and three quarters of students  **4.** Between three quarters and all students | **1**. Less than or around one quarter of students  **2**. Between one quarter and a half of students  **3.** Between one half and three quarters of students  **4**. Between three quarters and all students |

- 1. Thinking about all of the district’s efforts around physical activity, could you please tell us more about how the program(s) started? Where did the idea(s) come from? ___________________________________________________________________________________________________________________________________________________________________________________________________________________________________________________________________________________________________________________________________________________________________________________________________________
  2. What were your estimated program startup costs, if any? **[If none, continue to 3e]** _______________________________________________________________________________
  3. Were there any barriers to procuring startup funding for your program(s)? If so, what were the biggest challenges to obtaining funding? How did your district overcome these challenges? ___________________________________________________________________________________________________________________________________________________________________________________________________________________________________________________________________________________________________________________________________________________________________________________________________________
  4. Was funding required to start your program(s) **[circle one]**?
     1. No **[If no, continue on to question 4]**
     2. Yes
        1. If yes, is physical activity program funding a part of the district’s regular budget **[circle one]?**
           1. No
           2. Yes
        2. If yes, what was/were the funding source(s) when your program(s) started **[circle all that apply and provide written responses where applicable]**?
           1. Internal

Parent Teacher Association/Parent Teacher Organization

School Department budget

Physical Education budget

Other:__________________________________________________________________________________________

- - - - 1. External **[provide external funding source(s) in the space below]** ___________________________________________________________________________________________________________________________________________________________________________
  1. Is funding required to run the program(s) on an ongoing basis (year to year) **[circle one and provide written responses where applicable]**?
     1. No **[If no, continue on to question 3.g.]**
     2. Yes **[If yes, continue to sub questions 3.f.ii. 1, 2, and 3]**
        1. Where is the ongoing (year to year) internal, district funding currently coming from?
           1. Parent Teacher Association/Parent Teacher Organization
           2. Other:_____________________________________________________________________________________________________________
        2. Has funding for ongoing program costs been formally incorporated into the district’s annual budgets? ______________________________________________________________________________________________________________________________________________________________________________________________________________________________________________________________________________________________________________________________________________________________________________________________________
        3. Is there currently external funding from outside the district that supports the program? If yes, who is providing the funding, how much is provided, and for how long will your district be receiving the funding? _____________________________________________________________________________________________________________________________________________________________________________________________________________________________________________________________________________________________________________________________________
           1. **[PROBE]** Do you have funding from a PEP grant, community-based funding, or other school-based physical activity grant? __________________________________________________________________________________________________________________
  2. How many of your programs rely on volunteers or parents versus paid staff? _________________________________________________________________________________________________________________________________________________________________________________________________________________________________________________________________________________________________________________________________________________________________________________________________________________________________________________________________________________________________________________________________________________________________________

1. **Leadership:** We want to know more about the people/groups involved in the creation, implementation, and/or sustainability of the physical activity program(s) in your district. Specifically, who would you identify as the champions at the district level? A champion is an individual who is or a group of individuals who are instrumental in the development, implementation, and/or continuation of the physical activity program(s) in your district. Champions often provide the skills, experience, and/or enthusiasm necessary to encourage and maintain positive changes to physical activity programs. Please identify all the champions that apply from the following and note the number of individuals/groups in each category **[check all that apply and indicate the number of individuals. If applicable, provide the title of the champion(s) not included in the list below in space available in 2.j.**]**:**
   1. Physical Education teachers? ………………………….……..……………….…………………………...…….. **☐ ___**

**Number**

- 1. Principals? ………………………………………………………………………………………………………..………... **☐ ___**
  2. Family or Parent(s)?……………………………….…….……………………………………………………..……... **☐ ___**
  3. Board of Education/School Committee member?.…………………………………………………...... **☐ ___**
  4. Superintendent?…………………………………………………………………………………….…………………… **☐ ___**
  5. Community member or organization?………………………………………………………………………… **☐ ___**
  6. Parent Teacher Association or Parent Teacher Organization?…………………………..…………. **☐ ___**
  7. Director of Health and Physical Education? ……………………………….………………….……………. **☐ ___**
  8. Mayor’s office/County supervisor/Town Manager? ………….………………………………..………. **☐ ___**
  9. Other: _________________________________________________________________ **☐ ___**

1. **Sustainability** The following questions review the sustainability of the physical activity programmatic activities in your district.
   1. What factors might positively affect the sustainability of these program(s)? What factors might negatively affect the sustainability of the program(s)? __________________________________________________________________________________________________________________________________________________________________________________________________________________________________________________________________________________________________________________________________________________________________________________________________________________________________________________________________________________________
   2. Have physical education and physical activity requirements and performance measures been integrated into your local district’s school wellness policies? ___________________________________________________________________________________________________________________________________________________________________________________________________________________________________________________________________________________________________________________________________________________________________________________________________________
   3. Does your district have plans to sustain your programs past a certain time frame **[circle one and provide written responses]**?
      1. No. If no, why? ________________________________________________________________________________________________________________________________________________________________________________________________________________________________________________________________________________________________________________________________________________________________________
      2. Yes. If yes, for how long? ________________________________________________________________________________________________________________________________________________
2. **Implementation**: Is your district assessing whether your program(s) is/are being implemented as intended? Do you have any quantitative or qualitative data on the implementation process? _________________________________________________________________________________________________________________________________________________________________________________________________________________________________________________________________________________________________________________________________________________________________________________________________________________________________________________________________________________________________________________________________________________________________________________________________________________________
3. **Baseline/Outcome Evaluation:** Are any program outcomes being systematically assessed? Do you have any quantitative or qualitative data on outcomes from the program(s)?

____________________________________________________________________________________________________________________________________________________________________________________________________________________________________________________________________________________________________________________________________________________________________________________________________________________________________________________________________________________________________________________________________

1. **Equity**: Research suggests that disparities in obesity rates exist based on socioeconomic status, ethnicity, locality, disability, and gender. How do you think the district’s physical activity program strategy will affect the inequitable distribution of obesity in relation to these factors?

__________________________________________________________________________________________________________________________________________________________________________________________________________________________________________________________________________________________________________________________________________________________________________________________________________________________________________________________________________________________________________________________________________________________________________________________________________________________

1. **Policy Impact:** To what extent have the results of your physical activity program(s) affected the physical activity policies in your district? To what extent have local and/or state policies around physical activity in schools influenced the policies in your district? ___________________________________________________________________________________________________________________________________________________________________________________________________________________________________________________________________________________________________________________________________________________________________________________________________________________________________________________________________________________________________________________________________
2. **Final questions:** **[Ask only if the district has an implementation and outcome evaluation plan.]** Would you be open to sharing any data you have available? Would you or someone else at your district be willing to participate in a follow up interview? ________________________________________________________________________________________________________________________________________________________________________________________________________________________________________________________________________________________________________________________________________________________________________________________________________________________________________________________________________________________________________________________________________________________________________________________________________________________________________________________________________________________________________________
3. **Final questions: [Ask all interviewees.]** Do you have additional information or thoughts to share? ______________________________________________________________________________________________________________________________________________________________________________________________________________________________________________________________________________________________________________________________________________________________________________________________________________________________________________________________________________________________________________________________________________________________________________________________________________________________________________________________________________________________________________________________________________________________________________________________________

**Thank you again for taking the time to complete our interview. We are very appreciative of your time and are excited to learn about all of the activities your district is doing to keep kids active throughout the school day.**
