# Supplementary material for: Investigating best practices of district-wide physical activity programmatic efforts in US schools– a mixed-methods approach
Source: BMC Public Health. 2018 Aug 30;18:992. doi: 10.1186/s12889-018-5889-4 (PMC6117892; doi:10.1186/s12889-018-5889-4)
Supplement: Supplementary file 2 — Table S1. Demographic characteristics of participating versus non-participating districts (n = 59)a,b. Table S2. Comparison of physical education and physical activity program characteristics of nationally representative samples from the SHPPS 2006, 2012, and 2016 reports. (DOCX 16 kb) [file 12889_2018_5889_MOESM2_ESM.docx]

**Additional file 1**

**Table S1:** Demographic characteristics of participating versus non-participating districts (n=59)^a,b^

|  | Participating districts (n=23) | Non-participating, nominated districts (n=36) |
| --- | --- | --- |
| Geographic location (%)^b,c^ | | |
| Urban | 56 | 50 |
| Suburban | 39 | 28 |
| Rural | 4 | 22 |
| Socio-economic status (%)^b,d^ | | |
| Low SES | 39 | 22 |
| Middle SES | 48 | 67 |
| High SES | 13 | 11 |

^a^Demographic data of districts were compared when data were publically available.^14^

^b^P-values were generated from Fisher’s exact test, for cells containing expected counts <5

^c^P-value = 0.18

^d^P-value = 0.34

**Table S2:** Comparison of physical education and physical activity program characteristics of nationally representative samples from the SHPPS 2006, 2012, and 2016 reports

|  | 2006 | 2012 | 2016 |
| --- | --- | --- | --- |
| *PE requirements and offerings, overall and in relation to recommendations* | | | |
| Districts with a minimum PE requirements for students (%) |  |  |  |
| Elementary | 76 | 78 | 74 |
| Middle | 76 | 72 | 71 |
| High | 82 | 80 | 76 |
| Districts that require recess for students (%) |  |  |  |
| Elementary | 57 | 59 | 65 |
| Districts that offer school-based PA programs (%) |  |  |  |
| Elementary | 41 | 45 | 60 |
| Middle | 34 | 34 | 46 |
| High | 12 | 14 | 29.8 |

Results from the nationally representative sample of districts were abstracted and calculated from the Centers for Disease Control’s School Health Policies and Practices Study (SHPPS) for 2016 and 2012

^10^ and were abstracted from published results for the 2006 SHPPS study^12^.
